# Yiddish Language and Ashkenazic Jews: A Perspective from Culture, Language, and Literature

**DOI:** 10.1093/gbe/evw131

**Published:** 2016-06-11

**Authors:** Marion Aptroot

**Affiliations:** Abt. für Jiddische Kultur, Sprache und Literatur, Institut für Jüdische Studien, Heinrich-Heine-Universität Düsseldorf, Germany

**Keywords:** Yiddish, Ashkenaz, Ashkenazic Jewry

## Abstract

The typology of Yiddish and the name Ashkenaz cannot serve as arguments to support the theory put forward by Das et al. (2016). (Localizing Ashkenazic Jews to primeval villages in the ancient Iranian lands of Ashkenaz. *Genome Biol Evol*. 8:1132–1149.) that the origin of Ashkenazic Jews can be located in ancient Iran. Yiddish is a Germanic, not a Slavic language. The history of the use of the term Ashkenaz from the Middle Ages onward is well documented. Ashkenazic Jewry is named for the Hebrew and Yiddish designation for Germany, originally a Biblical term.

Recently in these pages, a paper by Das et al. (2016) appeared in which genetic comparisons among human individuals were undertaken that purportedly localize the origin of Ashkenazic Jews to primeval villages in ancient Iran. Though the experimental work was genetic, the content and potential implications of the paper crossed many disciplines, in particular the cultural and geographical history of the people whose ancestors spoke Yiddish. Any peer-reviewed journal which publishes interdisciplinary research is well advised to consult reviewers from the disciplines involved. It is possible that experts for Yiddish historical linguistics were not consulted in the peer review process. Several issues concerning Yiddish that were not discussed in the paper deserve attention.

Das et al. create a narrative based on genetic, philological and historical research and state that the findings of the three disciplines support each other. This is not the place to go into philological detail or to analyze the wide-ranging debates on the origins and development of Yiddish, but the bold statements Das et al. make about the Yiddish language and the name Ashkenaz cannot be left uncommented.

One of the central premises of the article is that Yiddish spoken in Eastern Europe was not a Germanic but a Slavic language. That idea was first put forward by Paul Wexler, one of the co-authors of the article mentioned above, in 1991. The *International Journal of the Sociology of Language* devoted a whole issue to Wexler’s original theory, giving him ample space to set out his idea that Yiddish is a relexified Slavic language and to reply to rebuttal articles (Wexler 1991). According to Wexler, larger numbers of Sorbians were to have converted to Judaism and two (partial) language shifts led to the creation of a language with a German vocabulary and Slavic grammar. Eastern Yiddish has indeed some characteristics of Slavic languages on all structural levels (lexicon, morphology, syntax, and phonology). However, not only is the bulk of the vocabulary of Germanic origins, its grammar is overwhelmingly Germanic, too: the conjugation of verbs, the case system, the use of definite and indefinite articles, the plural formation of most nouns, and the declension of adjectives. This is not an exhaustive listing, but it should suffice to explain in general terms that, typologically, Yiddish is a Germanic language. As has been pointed out before (Comrie 1991), Wexler dismissed this linguistic evidence by positing that syntax—which in the case of Yiddish has characteristics of both Germanic and Slavic as well as other contact languages—is the main factor determining linguistic typology. This is not a view shared by other linguists, who typically try to account for all secured information. Wexler’s theory did not gain acceptance in the scholarly community.

Das et al. state that Jews of Turkic origins settled in Eastern Europe and gave Ashkenazic Jewry its name. As evidence they cite three place names in Northern Turkey which sound somewhat like “Ashkenaz” as well as the story of the conversion of Khazars, inhabitants of a Turkic empire, to Judaism (on Khazars and Judaism, see Stampfer 2013). Weinreich’s model (Weinreich 1973, 2008), rejected by Das et al., also links Yiddish with Ashkenazic Jewry, but quotes textual sources for the use of the name Ashkenaz in post-Biblical times. In late Antiquity and the Middle Ages, scholars made attempts to link the names of the peoples mentioned in the Bible with contemporary geographical knowledge. In the Jewish and Christian traditions, Ashkenaz—a descendant of Noah’s son Japheth—is considered to be the ancestor of the peoples who lived to the North of Biblical Israel. The term Ashkenaz (which occurs in the Bible in Genesis 10: 3, 1 Chronicles 1: 1–5, and Jeremiah 51: 27) is used in Hebrew and Yiddish sources from the High Middle Ages onward to denote a region in what is now roughly Southern Germany. Jewish immigrants in Europe transferred Biblical names onto the regions in which they settled, for example, Ashkenaz for Southern Germany and Sepharad (Obadiah 20) for Spain. Any region north of Biblical Israel could have been named Ashkenaz. Within the Jewish world, where international correspondence between the main centers of learning in Europe, Asia, and North Africa was not a rarity, the region in the German lands where Jews had settled became known as Ashkenaz.

Wherever Jews lived, their rites and customs developed in a specific way due to cultural contact with local populations and—mainly through texts—with Jews elsewhere. With the spread of the customs and rites of the Jews of Ashkenaz over large parts of Europe, major centers of Ashkenazic Judaism had emerged in Eastern Europe by the 16th century.

The whole region in which the Ashkenazic cultural-religious complex dominated among Jews became known as Ashkenaz. However, as a more precise geographical term, Ashkenaz was still used to refer to Germany. This is evident in written references to the countries where Ashkenazic Jews lived, for example on the title-pages of prayer books and countless other printed works in Hebrew and Yiddish that were intended for the whole Ashkenazic world. The title-page of a 16th century Ashkenazic book of religious customs in Yiddish (Tyrnau 1589) states the book contains the religious customs in use throughout Germany (*in gants Ashkenaz*) and among Ashkenazim in Italy (*Velsh-land*), as well as the customs in Moravia (*Meyrern* [*Meyrn*]), Bohemia (*Peym*), and Poland (*Poyln*). Yiddish polemical poems and plays in which Polish and “Ashkenazic” (i.e., German) Jews use stereotypes and dialect features to make fun of each other have come down to us from the 16th century onward (e.g., Shmeruk 1979; Weinreich 1929; Aptroot 2010).

The distribution of Yiddish books and the polemics across Europe were possible not least because Jews in Western and Eastern Europe had a written language in common and spoke distinct dialects which were mutually intelligible to a high degree. The linguistic and cultural geography of Ashkenaz—the wider region where Jews following Ashkenazic customs and rites and their descendants once lived—cannot be restricted to certain parts of Eastern Europe, as Das et al. do.

The idea that Ashkenazic Jews in Eastern Europe were not necessarily descendants of Jews who lived in Germany in the High Middle Ages could explain the purported “exceptional growth” in population numbers among this group in Eastern Europe. Incomplete and unreliable data from times when people were not counted regardless of sex, age, religion or financial or social status on the one hand, and the dearth of linguistic evidence predating the 15th century on the other, leave much room for conjecture and speculation. Linguistic evidence, however, does not support the theory that Yiddish is a Slavic language, and textual sources belie the thesis that the name Ashkenaz was brought to Eastern Europe directly from a region in the Near East.

Although the focus and methods of research may be different in the humanities and the sciences, scholars should try to account for all evidence and observations, regardless of the field of research. Seen from the standpoint of the humanities, certain aspects of the article by Das et al. fall short of established standards.
